# Using forecasting to evaluate the impact of COVID‐19 on passenger air transport demand

**DOI:** 10.1111/deci.12549

**Published:** 2021-10-17

**Authors:** Xishu Li, Maurits de Groot, Thomas Bäck

**Affiliations:** ^1^ Leiden Institute of Advanced Computer Science Leiden University Leiden CA Netherlands

**Keywords:** COVID‐19, passenger air transport, airline recovery, demand forecasting, simulation

## Abstract

The COVID‐19 pandemic caused a drastic drop in passenger air transport demand due to two forces: supply restriction and demand depression. In order for airlines to recover, the key is to identify which force they are fighting against. We propose a method for separating the two forces of COVID‐19 and evaluating the respective impact on demand. Our method involves dividing passengers into different segments based on passenger characteristics, simulating different scenarios, and predicting demand for each passenger segment in each scenario. Comparing the predictions with each other and with the real situation, we quantify the impact of COVID‐19 associated with the two forces, respectively. We apply our method to a dataset from Air France–KLM and show that from March 1st to May 31st 2020, the pandemic caused demand at the airline to drop 40.3% on average for passengers segmented based on age and purpose of travel. The 57.4% of this decline is due to demand depression, whereas the other 42.6% is due to supply restriction. In addition, we find that the impact of COVID‐19 associated with each force varies between passenger segments. The demand depression force impacted business passengers between age 41 and 60 the most, and it impacted leisure passengers between age 20 and 40 the least. The opposite result holds for the supply restriction force. We give suggestions on how airlines can plan their recovery using our results and how other industries can use our evaluation method.

## 1 INTRODUCTION

The COVID‐19 pandemic caused many industries enormous losses. The aviation industry is one that was hit the hardest (Hollinger, 2020). According to the International Air Transport Association, passenger air transport measured as revenue passenger kilometer was down 90% year‐on‐year in April 2020 and still down 70% in August 2020. Till November 2020, most fleets were still grounded. However, the industry urgently needs to plan for recovery. Our research is conducted in cooperation with the fourth largest airline in Europe, Air France‐KLM. The initial question from the airline is how to recover effectively and efficiently from the COVID‐19 pandemic. To answer this question, the airline needs to identify the forces of COVID‐19 they are fighting against, because the effect of a recovery plan depends on whether it directly addresses the problem at source.

Current recovery plans of airlines include seeking aids and financial supports from governments, cutting capacity to rein in costs, implementing in‐flight service changes, such as enhanced measures for cleaning airplanes (Peterson, 2020), to rebuild passenger confidence and trust, and focusing on passenger retention, for example, by offering booking incentives (Albers & Rundshagen, 2020; Amankwah‐Amoah, 2020). Air France‐KLM is concerned with two strategies: cutting capacity and offering booking incentives. A potential danger of cutting capacity is that it will further restrain demand. For offering booking incentives, many airline managers believe that it only has values if the main reason why passengers choose not to fly is because they have a low willingness to fly. If it is not the case, offering booking incentives only lowers the profit as passengers who want to fly will do so even if there are no booking incentives. To investigate the effectiveness of these two recovery strategies, Air France‐KLM needs to investigate the impacts of flight route restrictions and low willingness to fly on demand, respectively.

Not only to airlines, but also to other industries, pandemics affect business demand in multiple ways and they are qualitatively different from typical disruptions. Craighead et al. (2020) illustrate that a typical disruption reshuffles the proverbial deck regarding supply and demand — often affecting one, but not the other. However, during the COVID‐19 pandemic, the force of disruption is strong enough to force extreme shifts in both demand and supply. As such, pandemics require scholars to take a fresh look at supply chain phenomena to help firms better prepare for the next pandemic and foster resiliency. By separating the forces of the pandemic and evaluating the impact of each force, firms can identify the salience of their issues and better allocate their limited resource for a swift recovery.

In the context of passenger air transport, although the damage of the pandemic is straightforward, that is, a drastic drop in demand, there are two forces impacting demand. First, COVID‐19 restricts supply. Passengers cannot travel because of the restrictions imposed by governments. Second, COVID‐19 depresses demand. Passengers' desire or need to travel naturally drops in times of pandemic (De Vos, 2020). The impacts of the two forces on demand can be different. In addition, given the same force, the impact can differ between passengers and flight routes. For example, considering supply restriction, COVID‐19 impacted passengers in Europe more than passengers in United States because passenger flights in Europe are mostly international and most of the travel restrictions are also international. If COVID‐19 has little impact on passenger willingness to fly on certain routes, airlines could expect a V‐shaped recovery once they resume operations on these routes. In this situation, instead of launching a marketing campaign for these routes, airlines should move their resource to other critical operations areas. If there is an impact on passenger willingness to fly and the impact varies between passengers, airlines should identify the segments of passengers who are affected the most, and launch targeted promotions.

In this article, we propose a method for separating the forces of the COVID‐19 pandemic based on the target area of the force and evaluating the respective impact on demand for passenger air transport. Our method consists of four steps. First, we divide passengers into different segments based on passenger characteristics (age and purpose of travel, or tier in the airline loyalty program and length of flight). Second, based on the availability of data and the scope of the problem, we select candidate models for predicting demand for each passenger segment. Third, we simulate two scenarios for the *pandemic* period (from March 1st to May 31st, 2020). The first scenario is the *business as usual* scenario in which we assume that there is no pandemic and the prediction here represents demand in a normal situation, considering passengers' behavior pattern. The second scenario is the *pandemic* scenario in which we consider the travel restrictions in reality by making specific flight routes unavailable. The prediction here represents demand in a situation where passengers follow their behavior pattern under the impact of specific travel restrictions. Fourth, we test the candidate forecasting models and apply the best performing model to predict demand for each passenger segment in each scenario. Comparing the prediction in the *business as usual* scenario with the real situation, we derive the twofold impact of COVID‐19 on demand for each segment. Comparing the prediction in the *business as usual* scenario with the prediction in the *pandemic* scenario, we derive the impact of COVID‐19 associated with supply restriction. Comparing the prediction in the *pandemic* scenario with the real situation, we derive the impact of COVID‐19 associated with demand depression.

We apply our method to a dataset from the joint loyalty program of Air France–KLM, which contains travel data of 5.8 million passengers and 51 million flights from June 1st, 2018 to May 31st, 2020. Our results show that in the *pandemic* period, COVID‐19 caused the airline a demand decline of 40.3% on average for passengers segmented based on age and purpose of travel. The 57.4% of this decline is due to demand depression, whereas the other 42.6% is due to supply restriction. The result that a large portion of the demand decline is due to supply restriction suggests a promising recovery once Air France–KLM fully resumes its operations. In addition, we find that the impact of COVID‐19 associated with each force varies between passenger segments. The force associated with demand depression impacted passengers between age 41 and 60 and travel mostly for business (*middle‐age & business*) the most, and it impacted passengers between age 20 and 40 and travel mostly for leisure the least. The opposite result holds for the force associated with supply restriction. Specifically, for the *middle‐age & business* segment, the demand depression impact of COVID‐19 counts for 97.8% of the total decrease, whereas the supply restriction impact of COVID‐19 only counts for 2.2%. Based on our results, we suggest the airline focus on resuming flights for passenger segments of which demand decline is mainly due to the supply restriction impact, and focus on restoring passenger confidence for passenger segments of which demand decline is mainly due to the demand depression impact. We also provide guidelines for other industries to use our method.

Our research contributes to the theory and practice in three ways. First, we use forecasting to evaluate the impact of a past special event, COVID‐19, on transport demand. Current evaluations on the impact of COVID‐19 are mostly based on direct comparisons between actual and historical values, for example, comparing the number of flights now with the number in the same period last year. This type of results may not give the most accurate picture of COVID‐19 because demand may increase or decline over time due to normal factors such as economic outlooks. Second, we quantify the impact of COVID‐19 on demand, based on whether it is associated with the travel restrictions or the depression on passenger willingness to fly. Current research on evaluating the impact of COVID‐19 does not measure the impact of each force of COVID‐19. Without properly identifying the forces firms are fighting against, recovery strategies may not be effective. Last, we consider differences between passengers when evaluating the impact of COVID‐19. Different passenger segments respond to economic or social events differently due to particular characteristics of the segments. Thus, evaluations that do not model segments separately may over‐ or underestimate the impact on a specific segment and a uniform recovery plan may not work for all segments.

The remainder of the article is structured as follows. In Section 2, we discuss the related research. In Section 3, we elaborate on our method. In Section 4, we apply the method to the *Flying Blue* dataset from Air France‐KLM. In Section 5, we present the results in our industry example. In Section 6, we give suggestions on how airlines can plan their recovery using our results and how other industries can use our method.

## 2 LITERATURE REVIEW

Our research is related to two streams of literature. First, it is related to transportation research on traffic flow prediction. Second, it is related to research on crisis and disaster management, particularly the study of operations management issues in managing epidemic outbreaks. In the transportation literature, various methods have been used for traffic flow prediction. Below our review is limited to urban traffic flow prediction and passenger air transport demand forecast.

Traffic flow prediction is dealt with at different time horizons. Long‐ and medium‐term forecasts, which usually have a 1‐ to 10‐year planning horizon, provide key inputs for infrastructure planning decisions such as freeway capacity planning. Short‐term forecasts, of which planning horizons vary from 1 h to 1 month, provide key inputs for daily operation management decisions such as congestion control. Regardless of the forecast horizon, traffic conditions in a transportation network are related to its previous patterns. Thus, transport volumes and other information are recorded at regular time intervals and time series models are often used for traffic flow prediction. Traditional time series models include linear stationary models such as autoregressive (AR) and moving average (MA), and linear nonstationary models such as AR‐integrated MA (ARIMA). ARIMA model is the most representative time series model used in the transportation industry for traffic flow prediction. Lee and Fambro (1999) compared the performance of four time series models for short‐term freeway transport volume forecasting. Their results showed that ARIMA model gave the most stable and accurate results for their industry example. Multivariate time series models, such as vector ARMA and space‐time ARIMA (Kamarianakis & Prastacos, 2003, 2005), have also been used for traffic flow prediction. To capture seasonal patterns in traffic data, Williams et al. (1998) proposed seasonal ARIMA (SARIMA) models.

In addition to time series models, machine learning algorithms such as artificial neural networks and support vector regression have been used for traffic flow prediction (Smith, 1994; Dia, 2001; Vlahogianni et al., 2005; Kumar et al., 2013; Lv et al., 2014). These algorithms are trained to learn a function between a high‐dimensional set of features and the target to be predicted. Machine learning algorithms are mostly used for real‐time urban traffic forecasting with a swift planning horizon, for example, from 15 to 40 min. The goal is to provide travelers the ability to choose better routes and provide authorities the ability to manage the transportation system in real time (Polson & Sokolov, 2017). Despite the superiority of machine learning algorithms in capturing spatial‐temporal relations and nonlinear effects, time series models show robust and accurate predictions in many real‐world applications with short‐ and medium‐term forecast horizons. For example, Lippi et al. (2013) presented an experimental comparison of different time series models and supervised learning models. They found that SARIMA model coupled with a Kalman filter is the most accurate model for short‐term traffic flow prediction.

In the air transportation industry, time series models are most commonly used for demand forecast. Samagaio and Wolters (2010) used AR and exponential smoothing models to make long‐term forecasts for the total number of passengers at Lisbon airport. Xie et al. (2014) developed a hybrid model based on seasonal decomposition and support vector regression for short‐term forecasting of air passenger at airports. Nai et al. (2017) proposed a hybrid model based on empirical mode decomposition and SARIMA for short‐ and medium‐term air traffic forecasting.

The first limitation of the current traffic flow prediction practices is that they often underestimate the impact of characteristics of passengers on their travel behavior. Bhaskar et al. (2014) pointed out that the majority of studies in public transport neglect differences between groups of passengers. In addition, the existing passenger segmentation methods are limited to the use of passenger surveys. Although passengers' stated preference is valuable for transport demand forecasting (Park & Ha, 2006), segmentation can rely on passengers' exhibited behavior pattern (Bhaskar et al., 2014; Briand et al., 2017). Air passengers are known for their distinct characteristics; thus, to obtain accurate demand forecasts, the market should be segmented based on passenger characteristics and forecasting should be done for different segments separately. The second limitation of the existing transport demand forecasting models is that they often neglect the occurrence of large‐scale disasters. Li et al. (2017) argued that traditional traffic flow prediction methods focus on regular demand forecasting and have disadvantages in predicting passenger flows under special events scenario such as concerts and parades. Special events including large‐scale disasters have a disruptive impact on public transportation systems, and thus, should be given more attention in traffic flow prediction for proactive management. Comparing the forecast considering a special event with the real situation, the impact of the event on transport demand can be estimated and it helps governments and firms plan effective recovery strategies.

In the disaster operations management (DOM) literature, among all types of disasters that are studied, epidemics is rarely the focus. Altay and Green III (2006) surveyed the operations research literature on DOM. They found that only 11.9% of the existing studies focus on natural disasters, whereas the majority is on man‐made emergencies such as industrial accidents, spills, and computer network crashes. They outlined four stages of DOM, that is, mitigation, preparedness, response, and recovery, and found that the one that is in dire need of more research is disaster recovery. Disasters like a pandemic are difficult and costly to mitigate and prepare. The focus should, without question, be on response and recovery. To effectively and efficiently recover, the key is to identify the source of the problem and its impact on business. Pournader et al. (2020) conducted a review of supply chain risk management (SCRM) articles between 2001 and 2019. They found that little attention has been given to studies on business continuity and resilience management or humanitarian operations and disaster relief, compared to other more popular SCRM topics. Zhu et al. (2004) proposed a framework for understanding the impact of service failures on customers and designing cost‐effective recovery strategies. They suggested that appropriate resource allocations for outcome and process recovery strategies should be based on customer risk profiles and the firm's cost structures.

Using interviews, Suau‐Sanchez et al. (2020) provided an early assessment of the medium‐ and long‐term impact of COVID‐19 on air transport in terms of supply and demand. Their interview results showed that there will be a consolidation trend in supply, especially in the European market, and demand will be highly affected, even in the long term, because of changes in passenger behavior. In addition, on the demand side, the interviewees expressed their concern for business‐related long‐haul travel. Although the results showed a depressing future for airlines, Suau‐Sanchez et al. (2020) recognized that their study does not consider recovery scenarios as the interviews were conducted during the first weeks of the crisis. If they were to consider recovery scenarios, the effect of these scenarios should be evaluated and it will require the researchers to measure the impact of COVID‐19 on demand more precisely. Using an online survey, Graham et al. (2020) studied the attitudes of aging passengers (defined as aged 65+) toward air transport in times of pandemic. The findings showed that over 60% of aging, passengers are planning to travel by air in the next 12 months. Factors such as flexible ticket booking and quarantine rules that are key drivers affecting travel decisions of other groups of passengers do not appear to be key drivers for aging passengers. Their study demonstrated the importance of evaluating the impact of COVID‐19 on different groups of passengers separately.

The limitation of the current DOM studies on the impact of COVID‐19 is that they lack quantitative analyses. It may be due to a lack of data as the impact of COVID‐19 is yet to unfold. Forecasting models based on historical data can be used to evaluate the impact of the ongoing pandemic. In addition, the current studies on the impact of COVID‐19 on the transportation sector do not consider passenger segmentation. Tirachini and Cats (2020) synthesized on research needs pertaining to contagion risk in public transportation. One urgent research need is on the assessment of passengers' behavioral responses and adaptations in the post lockdown phase. They pointed out that although COVID‐19 has negatively affected all passengers' desires to travel, the extent of the effect varies considerably, depending on personal preferences as well as household income. Therefore, research on predicting passengers' behavioral changes should consider the differences in people's willingness to travel and their ability to travel if they so desire.

Our research contributes to the literature in two ways. First, we contribute to the transportation literature on traffic flow prediction by forecasting transport demand in a travel restriction scenario. In addition, we segment passengers based their demographic and trip‐related characteristics and perform forecasting for different segments separately. Second, we contribute to the DOM literature by using forecasting to evaluate the impact of COVID‐19 on passenger air transport demand and quantify the impact based on whether it is associated with supply restriction or demand depression. In addition, we evaluate the impact of COVID‐19 for different groups of passengers separately.

## 3 METHODOLOGY

We evaluate the impact of COVID‐19 on demand for passenger air transport. Passenger air transport demand can be measured in many forms such as the number of scheduled passenger, the number of scheduled flights, passenger‐kilometer, and revenue passenger kilometers (Banerjee et al., 2020; Marazzo et al., 2010). In this research, we model different passenger segments separately and measure demand for a segment in the number of unique flights completed by each passenger in the segment. It is different from the number of flights planned or executed by an airline because the former is for each individual passenger, reflecting her/his willingness to fly, whereas the latter considers all passengers as a whole. Measuring demand at the individual level helps capture differences between passengers. In addition, flight scheduling considers many other factors such as competition for market share than demand based on passenger willingness to fly. We measure daily demand if not specified. Another important note on our demand measure is that in airline databases, flights information is registered on the flight‐leg^1^ level, instead of based on the origin destination on the ticket. For example, if a ticket is from Amsterdam (AMS) to Paris (ORY) with a layover at Brussels (BRU), this ticket will be registered as two separate flights in the database, that is, AMS‐BRU and BRU‐ORY.

Our evaluation method consists of four steps. First, we divide passengers into different segments based on passenger characteristics. Second, we select candidate models for predicting demand for each passenger segment. Third, we simulate two scenarios for the *pandemic* period. Fourth, we test the candidate forecasting models and apply the best‐performing model to predict demand for each passenger segment in each scenario. Below, we elaborate on each step.

### 3.1 Step 1. Passenger segmentation

The first step is to segment passengers using data till the *pandemic* period. The concept of market segmentation was proposed by Smith (1956) for accommodating the diversity or heterogeneity among customers and providing better marketing strategies. It has been widely used in the aviation industry for the same purposes, for example, see Park (2007), Mukhopadhyay et al. (2007), and Harrison et al. (2015). Passenger segmentation is conducted either by dividing passengers into predefined groups or by clustering methods. Clustering methods such as density‐based clustering help determine the number of segment based on the heterogeneity in the data (Kriegel et al., 2011). When a fixed number of segments is required, k‐means clustering can be used (Hartigan & Wong, 1979). A potential drawback of clustering methods is that the composition of each segment, as well as the number of segments, is determined endogenously, which might not be practical from the business perspective or might not generate actionable insights. In practice, if clustering results have proven to be robust in a specific industry, these clusters can be used to generate group labels and future segmentation can be done by dividing data into the predefined groups.

In the airline industry, passenger segmentation is usually done by dividing passengers into predefined groups based on passenger characteristics that have proven effective in explaining the heterogeneity in passenger behavior. To extend the applicability of our method to different airlines or other industries, in this research, we also choose this approach. Passenger characteristics that impact passengers' travel behavior include demographics information of passengers such as age and income level. Furthermore, membership in a tiered loyalty program, employment status, and whether passengers are emigrants emerge as important determinants of travel demand (Warburg et al., 2006; Adikariwattage et al., 2012; Kuljanin & Kalić, 2015; Cook et al., 2017). In addition to demographic characteristics, geographic characteristics such as origin location data (home postcodes) of passengers can be extremely useful for effective passenger segmentation and targeting (Leung et al., 2017). However, each airline usually has one targeted passenger region, from which more than half of its flights and passengers originate. For example, over 80% of Air France‐KLM flights depart from EU, among which over 50% depart from France and are for French passengers. Thus, if there is only data of one airline, geographic information may not be effective in passenger segmentation.

In addition to demographic and geographic characteristics, trip‐related information such as length of flight and travel motive can be used in passenger segmentation. For example, based on different travel motives, passengers can be divided into business or leisure passengers. Brons et al. (2002) found that leisure travel demand and business travel demand are fundamentally different as they are affected by different factors. Because of the essential differences between the two passenger groups, leisure and business travelers are likely to respond differently to changes in certain socioeconomic factors and to events like travel restrictions. Therefore, they should be considered separately when forecasting demand in a pandemic situation. According to the common practice in the airline industry, a trip is classified as a business trip and the passenger is classified as a business traveler if one of the following conditions is met: (1) the ticket is purchased from a corporation account or (2) the cabin class of the ticket is a business class. Considering passenger p's flight history in a given period, we calculate the percentage of the time the passenger flies on a business trip as follows:

(1) $$P_{p}=\frac{1}{A_{p}}\sum_{i=1}^{A_{p}}\max\left\{C_{i},K_{i}\right\},$$

where $A_{p}$ is the total number of flights passenger p takes; $C_{i}\in \left\{0,1\right\}$, where $C_{i}=1$ if the ticket for flight i is purchased from a corporate account and 0 otherwise; $K_{i}\in \left\{0,1\right\}$, where $K_{i}=1$ if the cabin class of the ticket for flight i is a business class and 0 otherwise.

When segmenting passengers, the number of desired segments is an important factor that airlines should consider and the more is not always better. Depending on the goal of passenger segmentation, for example, when it is to retain passengers rather than to reduce the cost of the existing passenger reward scheme, the value of fewer segments may outweigh that of an overly‐refined segmentation. In addition, with more passenger segments, it could become difficult to interpret the results and generate actionable insights.

### 3.2 Step 2. Forecasting model selection

The second step is to select candidate models for predicting demand for each passenger segment. The choice of a forecasting model depends on the availability of data and the scope of the problem. For short‐term forecasts with time series data, models such as AR, MA, and ARMA are often used. When time series data show evidence of nonstationarity, the ARIMA model is a good candidate model. When seasonality exists in time series data, the seasonal ARIMA (SARIMA) model can be used. As SARIMA model only requires univariate time series data, it can be used in a broad range of industry sectors. If time series exhibit complex and multiple seasonal patterns (e.g., a monthly pattern and an annual pattern), (Trigonometric seasonality, Box‐Cox transformation, ARMA errors, Trend and Seasonal components) TBATS model can be used. When multivariate data are available or there is a need for real‐time forecasting, multivariate ARIMA‐based models or more complex models such as artificial neural networks can be deployed. In our industry example in which univariate time series data exhibit nonstationarity and seasonality, we choose two candidate models: SARIMA and TBATS.

SARIMA model considers seasonality in the data by including additional seasonal terms in ARIMA model (Hamilton, 1994). The AR part of ARIMA indicates that the evolving variable of interest is regressed on its own lagged values. The MA part indicates that the regression error is a linear combination of error terms whose values occurred both contemporaneously and at various times in the past. The I indicates that the data values have been replaced with the difference between their values and the previous values. This differencing process can be performed multiple times to make the model fit the data as well as possible.

The seasonal part of the model consists of terms that are very similar to the nonseasonal components of the model, but they involve backshifts of the seasonal period.

TBATS is an acronym for key features of the model: T is for trigonometric seasonality, B is for Box‐Cox transformation, A is for ARIMA errors, T is for trend, and S is for seasonal components. TBATS model takes it roots in exponential smoothing methods (De Livera et al., 2011).

Before applying a candidate model to predict future demand, it is important to test the performance of the model using historical data. In the case where accuracy measurements show a poor performance of the model, alternatives should be selected.

### 3.3 Step 3. Scenario simulation

The third step is to simulate two scenarios for the *pandemic* period. The first scenario is the *business as usual* scenario, and the prediction in this scenario represents the number of flights that each passenger segment would take in a normal situation, assuming that passengers follow their behavior pattern. This prediction serves as a baseline and will be compared with the prediction in the other scenario and with the real situation. The second scenario is the *pandemic* scenario. In this scenario, we make specific flight routes unavailable according to the travel restrictions in reality, and the prediction here represents demand in a situation where passengers follow their behavior pattern under the impact of specific travel restrictions. We assume that passengers' flight route choice will stay the same as before when evaluating the impact of a flight route restriction on demand; thus, this impact is proportional to the previous frequency at which passengers flew on the route. We recognize that this is a strong assumption. However, since the *pandemic* period which we select in this research only lasts 3 months (from March 1st to May 31st, 2020) and it is in the early months of the pandemic, we expect that passengers' flight route choices based on their own willingness to fly will not change. This assumption helps separate the impact of COVID‐19 associated with supply restriction from the impact of COVID‐19 associated with demand depression. In addition, because our industry example is a market leader, instead of a budget airline, and because our data are collected from the early months of the pandemic, we assume that the flight route restrictions in the *pandemic* period are results of government regulations, but not because of passengers cancelling flights.

To simulate the travel restrictions that had taken place in the *pandemic* period, data about flight routes availability need to be collected. This could be done by collecting public information on the travel restrictions, for example, airline announcements on flight cancellations. However, this information is not trivial to collect. We can also collect travel restriction data following three steps: (1) retrieve the preplanned^2^ schedule for each flight in the *pandemic* period. Use $N_{r}$ to denote the number of days in the *pandemic* period for which a flight was scheduled on flight r. (2) Retrieve the executed flights for each flight in the *pandemic* period. Use $D_{r}$ to denote the number of days in the *pandemic* period for which a flight was executed on flight r. (3) Calculate the availability of flight r in the *pandemic* period as $\frac{D_{r}}{N_{r}}$.

Table 1 shows an example of the availability of six flights in a period of 4 days. In the table, the value “True” indicates that the flight is available on the specific date and this date is retrieved from the preplanned schedule for the flight. For example, on March 25th, 2020, a flight on route AMS ‐ BRU was supposed to take off and in reality a flight took off; thus, this route is marked as available (“True”) on this date. We calculate the availability of a flight route in the *pandemic* period based on the percentage of days this route is available.

**TABLE 1:** Example of availability in the dataset

Flight route	2020‐03‐25	2020‐03‐26	2020‐03‐27	2020‐03‐28	Availability
AMS ‐ BRU	True	True	False	False	1/2
AMS ‐ ORY	True	True	True	True	1
BRU ‐ AMS	True	True	True	False	3/4
BRU ‐ ORY	True	True	False	False	1/2
ORY ‐ AMS	False	False	False	False	0
ORY ‐ BRU	True	False	False	False	1/4

### 3.4 Step 4. Prediction comparison

The last step is to apply the best‐performing forecasting model to predict demand for each passenger segment in each scenario. We first obtain the prediction in the *business as usual* scenario, using historical demand for each segment. Comparing the prediction in the *business as usual* scenario with the real situation, we derive the twofold impact of COVID‐19 on demand for passenger air transport, assuming that passengers will follow their behavior pattern if there was no pandemic. The time series prediction uses univariate flight data and the availability of flight routes is not considered. To obtain the prediction in the *pandemic* scenario, we first identify passengers' behavior pattern in terms of flight route choice and then adjust the prediction in the *business as usual* scenario, considering the impact of each flight route restriction on demand. Not every flight route restriction has the same impact on demand. Restrictions on popular routes result in severer impact than restrictions on less‐popular routes. In addition, restriction on a specific flight route may impact demand for different passenger segments differently because not every segment will fly on the route with the same frequency.

The flight route choices of each passenger segment can be identified by calculating the frequency at which the segment flies on each flight route, using historical data. In the *pandemic* scenario, the impact of a route restriction on demand for a segment considers both the availability of the route and the previous frequency at which the segment flew on this route. For example, if a segment flew from AMS to BRU, 30% of the time previously and in the *pandemic* period flights on this route were canceled 90% compared to the previous schedule, then the impact of this travel restriction on demand for this passenger segment, measured in the number of flights reduced, will be equivalent to 30%×90% of the demand forecast in the *business as usual* scenario. The prediction in the *pandemic* scenario is given by Equation (2):

(2) $$F_{s}^{p}=F_{s}^{b}\sum_{r=1}^{R_{s}}\frac{A_{r|s}D_{r}}{A_{s}N_{r}}\;,$$

where $F_{s}^{p}$ is the forecast on the number of flights for passenger segment s in the *pandemic* scenario, $F_{s}^{b}$ is the forecast on the number of flights for passenger segment s in the *business as usual* scenario, $L_{s}$ is the number of flight routes on which passenger segment s has flown previously, $A_{r|s}$ is the number of flights on route r passenger segment s has flown previously, $A_{s}$ is the total number of flights passenger segment s has flown, and $\frac{D_{r}}{N_{r}}$ calculates the availability of flight route r in the *pandemic* period (see Step 3 of the method).

The impact of a flight route restriction on demand for a passenger segment can also be evaluated by directly multiplying the availability of this route with the demand forecast for this route. However, it will then require forecasting to be done on the flight route level, that is, demand forecasting for each flight route for each passenger segment. The potential disadvantage of this approach is that there may be a lack of time series data on the flight route level within a passenger segment, and thus the forecast may not be accurate. By aggregating the flight data on different flight routes, generating an overall demand forecast for the entire set of flight routes, and considering the weight of each flight route in the demand forecast, we maintain good accuracy of the forecasting model and extend the applicability of our approach to a broad range of industries.

In the *pandemic* scenario, the impact of the availability of flights on demand is considered. Comparing the prediction in the *business as usual* scenario with the prediction in the *pandemic* scenario, we derive the impact of COVID‐19 associated with supply restriction. Comparing the prediction in the *pandemic* scenario with the real situation, if there is a lower number of flights in the real situation, it can be attributed to a low willingness to fly. Therefore, the difference between the prediction in the *pandemic* scenario and the actual number of flights is the impact of COVID‐19 associated with demand depression.

## 4 APPLICATION TO THE *FLYING BLUE* DATASET

We apply our method to a dataset collected from the largest air passenger loyalty program in Europe, the *Flying Blue* program of Air France–KLM. The dataset contains passenger and flight specific data. Passenger‐specific data consist of the identification number of the passenger, the age of the passenger, and the membership information of the passenger. Flight‐specific data consist of the date of the flight, the purchasing account of the ticket, the cabin class of the ticket, the origin and destination of each leg of the fight, and the length of the flight. The flight data are linked to the passenger data, that is, for each passenger in the program, we can retrieve her/his previous flights. In addition, the passenger‐specific data are updated in real time, for example, for each flight, there is a data entry on the current age of the passenger and current tier at which the passenger is in the loyalty program. Table 2 provides a description of the dataset.

**TABLE 2:** Description of the data in the dataset

Type	Variable name (notation)	Description
Passenger specific data	CIN (I)	An unique identifier of the passenger in the loyalty program
	Age (A)	The age of the passenger on a specific date
	Tier (R)	The tier at which the passenger is in the loyalty program^3^
Flight specific data	Flight Date (T)	The date of the flight leg
	Corporate Purchaser (C)	Whether the ticket is purchased from a corporation account
	Cabin Class (K)	Whether the cabin class of the ticket is a business class
	Origin (O)	The origin airport of the flight leg
	Destination (D)	The destination airport of the flight leg
	Flight Length (M)	The miles of the flight leg

We left‐censor the flight‐specific data on June 1st, 2018 and right‐censor it on May 31st, 2020. March 11th is the date on which the World Health Organization (WHO) declared the coronavirus outbreak a pandemic, but some travel restrictions had already taken place before that date; therefore, in our analysis, we select the first date in March as the starting date of the *pandemic* period. The *pandemic* period ends on the right‐censoring date of the dataset, that is, May 31st, 2020. In total, the dataset contains data of 5.8 million passengers and 51 million flights. Table A1 in the Appendix lists the key dates in the dataset used in our research. We follow the four steps of our evaluation method. That is, we divide passengers in the dataset to different segments, simulate the two scenarios for the *pandemic* period, test the performance of the two candidate models, and apply the best‐performing model to predict demand for each passenger segment in each scenario and compare predictions. Below, we elaborate on passenger segmentation and model application.

### 4.1 Passenger segments in the *flying blue* dataset

Our methodology choices for passenger segmentation in the Air France‐KLM example are largely motivated by practice. The merits of such an approach are that results would be intuitive and practically easy to implement. In the airline industry, passenger segmentation uses both passenger‐ and flight‐specific data. Based the age of the passenger or the tier at which the passenger is in the loyalty program on the starting date of the *pandemic* period, passengers can be divided into three groups: *young/middle‐age/aging* or *explorer/silver/higher* passengers. Considering a passenger's flight history till the *pandemic* period, we calculate the percentage of the time the passenger flies on a business trip according to Equation (1). Based on this percentage or the average length of the passenger's previous flights, she/he is assigned to one of the following three groups: *leisure/middle‐class/business* or *short‐/medium‐/long‐haul* passengers. The criterion for each group and the distribution of passengers across each set of three groups are shown in Table 3. These criteria are selected based on our discussions with the airline managers at Air France‐KLM to ensure a relatively fair representation of all passengers and they meet the airline's estimations on the portion of each passenger group. The dataset also contains a small portion of passengers, around 3.8%, whose age is below 20. However, because teenagers usually depend on others, for example, their parents, when it comes to air travel decisions (Copperman & Bhat, 2007), we exclude this portion of passengers in our analysis. In addition, we exclude passengers whose age is above 80 since there is a large number of outliers in the time series flight data of this group, and this group is less than 1% of the total passengers in the dataset and counts for even a smaller portion of the total flights. As explained earlier, since we only have data of one airline, passenger geographic information or the origin of flight is not used here to segment passengers.

**TABLE 3:** Distribution of passengers in the dataset

Age		Purpose of travel	
*Young* (age 20–40)	31%	*Leisure* (business trip <20%)	51.9%
*Middle‐age* (age 41–60)	52.5%	*Middle‐class* (business trip 20%–70%)	18.9%
*Aging* (age 61–80)	16.5%	*Business* (business trip >70%)	29.2%
Tier		Flight Length	
*Explorer* (the entry tier)	61.4%	*Short‐haul* (<2000 miles)	27.5%
*Silver* (one tier higher than *Explorer*)	13.8%	*Medium‐haul* (2000–3500 miles)	39.6%
*Higher* (higher tiers than *Silver*)	24.8%	*Long‐haul* (>3500 miles)	32.9%

Following the suggestions of the airline managers at Air France‐KLM and for the purpose of generating actionable insights, we formalize nine passenger segments based on a passenger‐specific variable and a flight‐specific variable. Specifically, we divide passengers either based on age and purpose of travel or based on tier and flight length: *young/middle‐age/aging & leisure/middle‐class/business* (denoted as A1–A9) or *explore/silver/higher & short‐/medium‐/long‐haul* (denoted as B1–B9) passengers. The tier at which a passenger is in the loyalty program could be closely related to the passenger's purpose of travel, that is, the higher the tier, more likely the passenger travels for business. For example, among the *explorer* tier, 64.7% belong to the *leisure* passenger group and among the *higher* tier, 50.5% belong to the *business* passenger group. In addition, the age of the passenger and the average length of her/his flights could be closely related. Middle‐age passengers typically fly long hauls more often than the other passenger age groups, whereas aging passengers take the least amount of long hauls. Therefore, we do not segment passengers based on tier and purpose of travel or based on age and length.

**FIGURE 1:**
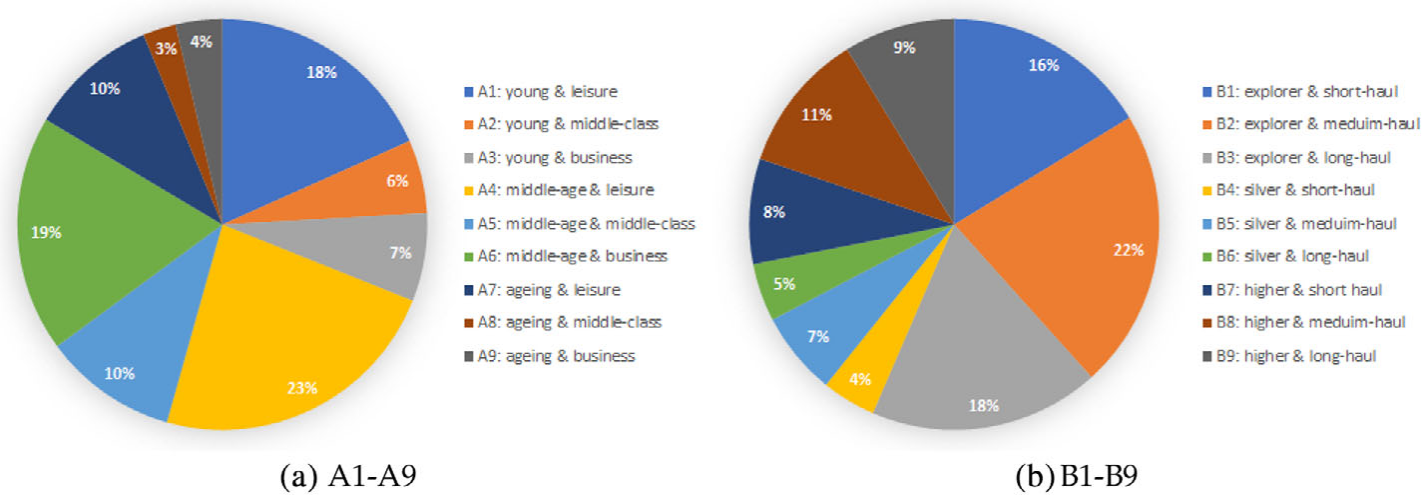
Distribution of passengers across A1–A9/B1‐B‐9 segments

**FIGURE A1:**
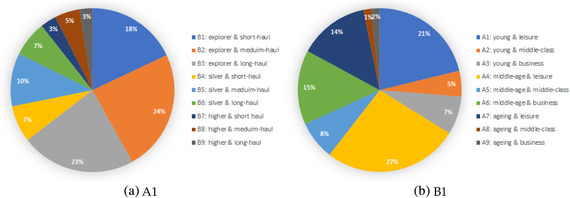
Distribution of A1/B1 passengers across B1–B9/A1–A9 segments

Table 4 presents the distribution of passengers in each group when using each of the two segmentation plans. The results show that in each segmentation plan, there are no correlations between groups based on passenger specific data and groups based on flight specific data. Figure 1 shows the passenger distribution across each set of nine segments. We also check whether the two sets of segments overlap, the results show no significant overlaps between any two segments. As two examples, Figures A1(a) and A1(b) in the Appendix show the distribution of A1 passengers across B1–B9 segments and the distribution of B1 passengers across A1–A9 segments, respectively. An ANOVA test (p<0.01) is performed to confirm that each set of nine segments exhibit different patterns among each other.

**TABLE 4:** Distribution of passengers in each group

Young		Middle age		Aging	
*Leisure*	55%	*Leisure*	41.4%	*Leisure*	59.2%
*Middle‐class*	21.1%	*Middle‐class*	21.4%	*Middle‐class*	17.2%
*Business*	23.9%	*Business*	37.2%	*Business*	23.6%
*Leisure*		*Middle‐class*		*Business*	
*Young*	33.4%	*Young*	30%	*Young*	22.6%
*Middle‐age*	46.2%	*Middle‐age*	56.1%	*Middle‐age*	64.7%
*Ageing*	20.4%	*Ageing*	13.9%	*Ageing*	12.7%
*Explorer*		*Silver*		*Higher*	
*Short‐haul*	28.9%	*Short‐haul*	27.7%	*Short‐haul*	29.5%
*Medium‐haul*	39%	*Medium‐haul*	42.7%	*Medium‐haul*	39.4%
*Long‐haul*	32.1%	*Long‐haul*	29.6%	*Long‐haul*	31.1%
*Short‐haul*		*Medium‐haul*		*Long‐haul*	
*Explorer*	56.5%	*Explorer*	55.5%	*Explorer*	57.7%
*Silver*	14.8%	*Silver*	16.6%	*Silver*	14.5%
*Higher*	28.7%	*Higher*	27.9%	*Higher*	27.8%

**FIGURE 2:**
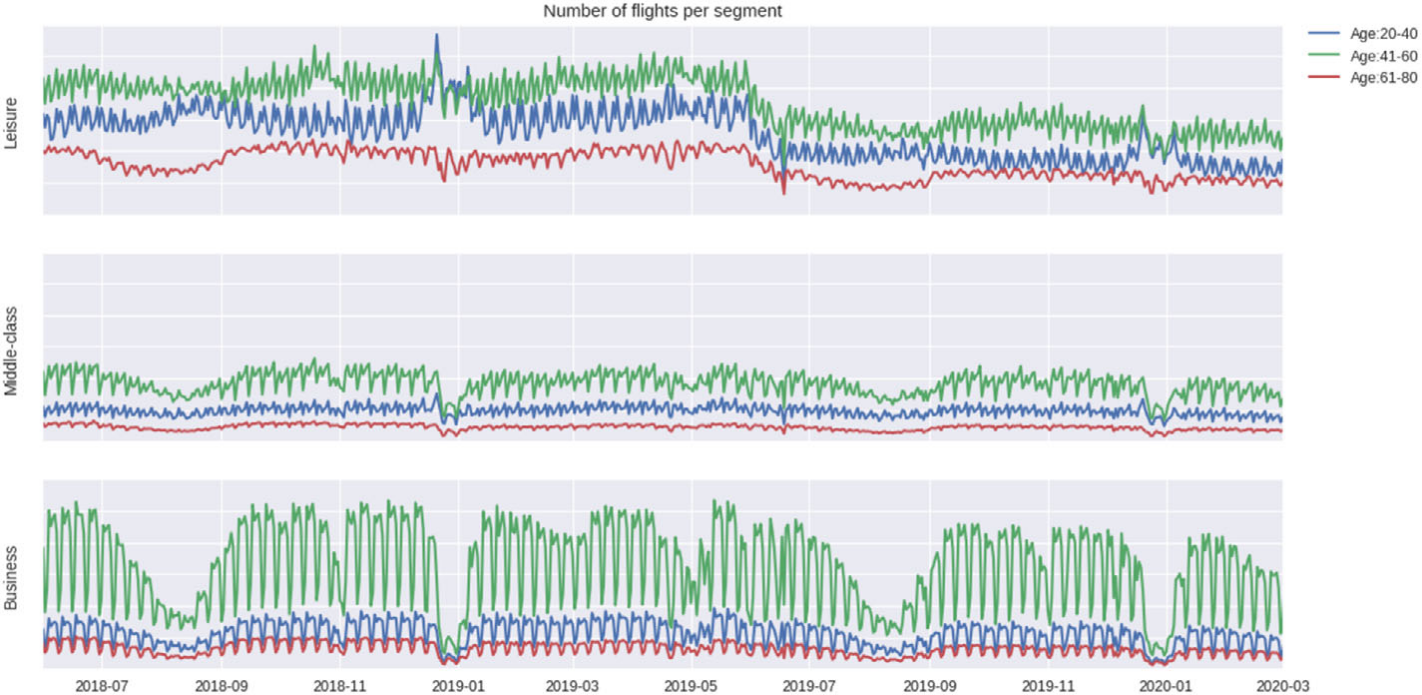
Behavior pattern of A1–A9 segments

**FIGURE 3:**
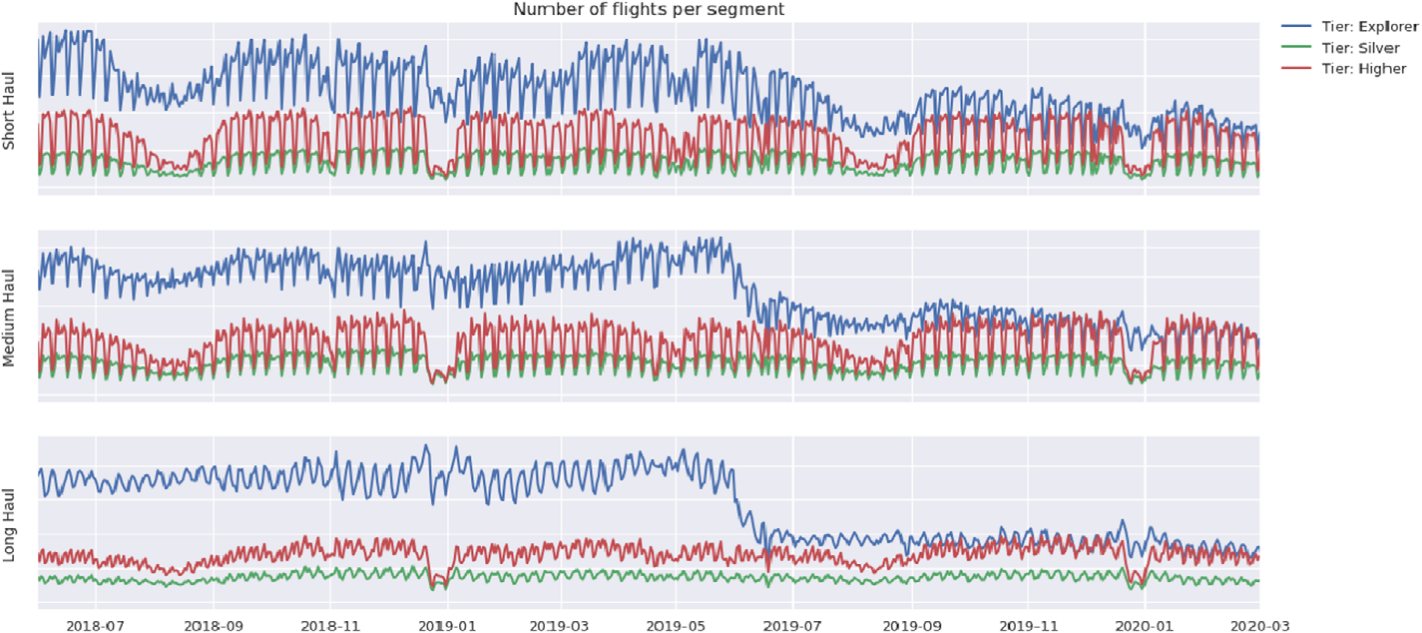
Behavior pattern of B1–B9 segments

Figures 2 and 3 present an overview of the 21‐month flight history (till the *pandemic* period) of A1–A9 and B1–B9 segments, respectively. The *y*‐axis shows the total number of flights for a passenger segment; thus, when comparing the three lines in a plot, the distribution of passengers should be considered. For example, in Figure 3, the *explorer* tier (the blue line) takes many more flights than the other two tier groups. This may be because that in each group based on flight length, over 55% of passengers are at the *explorer* tier (see Table 4). Due to the data privacy requirement of the airline, the units of flights (the *y* axis of all figures) are hidden in this research and the *y* axes of different figures are not on the same scale. However, comparing the lines of the same color in the three plots with each other, it is still evident that the passenger groups within the same age range behave differently if the purposes of their trips are different or that passenger groups at the same tier behave differently if the lengths of their flights are different. For example, in Figure 2, the level in a year of flight data for the *middle‐age & leisure* segment is relatively stable, comparing to that for the *middle‐age & middle‐ class* segment or the *middle‐age & business* segment. Comparing the three lines in each plot in Figures 2 and 3, it is also clear that different age groups with the same travel purpose or different tier groups with the same flight length behave differently.

Figures 2 and 3 also show evidence of nonstationarity and seasonality in the flight data for each passenger segment. For example, in Figure 2, the *middle‐class & leisure* segment and the *young & leisure* segment are flying less in 2019 (a turning point in July 2019) compared to 2018. For the majority of each set of nine passenger segments, clear seasonal patterns occur. For example, for the *young & leisure/business* segment and the *aging & leisure/middle‐class* segment in Figure 2, the number of flights drops in January and August each year. Because of these patterns in the time series data, a candidate model is SARIMA model. As there may exist multiple seasonalities, we also consider TBATS model.

### 4.2 The model selection and parameter estimation

To test the performance of each candidate model, we use the first 15 months of demand (from June 1st, 2018 to September 30th, 2019) for all passengers in the dataset. Using maximum likelihood estimation, autocorrelation plot and partial autocorrelation plot (the two plots are shown in the Appendix), the estimated parameters of SARIMA model are: (p,d,q)=(6,1,2), and (P,D,Q)=(0,1,1). The parameters of TBATS model are listed in Table A2 in the Appendix. The mean absolute percentage error (MAPE) and Akaike information criterion (AIC) associated with the two models are: MAPE of 0.1288 and AIC of 9294.475 for SARIMA model and MAPE of 0.1409 and AIC of 10819.972 for TBATS model. As the more complex model, TBATS model, does not improve the forecast performance, hereinafter, we apply SARIMA model to predict demand for each passenger segment in each scenario, using historical demand till the *pandemic* period (from June 1st, 2018 to February 29th, 2020).

We first obtain the prediction in the *business as usual* scenario. SARIMA model parameters for each passenger segment are listed in Table A3 in the Appendix. The prediction in the *pandemic* scenario depends on the prediction in the *business as usual* scenario and the impact of each flight route restriction on a passenger segment. To derive the impact of a flight route restriction, we calculate the availability of each flight route in the *pandemic* period (see Step 3 in Section 3 for how we collect travel restriction data). The average availability of 51 million flights is 0.3658 and Table 5 lists the average flight availability for each passenger segment. The results show that the extent of supply restriction, without considering the weight of each route in a segment's route choices, is similar between segments. Next, we identify flight route choices of each passenger segment, using historical data till the pandemic (from June 1st, 2018 to February 29th, 2020). Incorporating both the availability of each flight route and flight route choices of each passenger segment, we obtain the demand forecast in the *pandemic* scenario according to Equation (2).

**TABLE 5:** Availability of flight routes for A1–A9/B1–B9 segments

A1–A9 segments	Flight availability	B1–B9 segments	Flight availability
*Young & Leisure*	0.3754	*Explorer & Short‐haul*	0.3192
*Young & Middle‐class*	0.3792	*Explorer & Medium‐haul*	0.4434
*Young & Business*	0.3786	*Explorer & Long‐haul*	0.3142
*Middle‐age & Leisure*	0.3629	*Silver & Short‐haul*	0.3103
*Middle‐age & Middle‐class*	0.3657	*Silver & Medium‐haul*	0.4502
*Middle‐age & Business*	0.3767	*Silver & Long‐haul*	0.3201
*Aging & Leisure*	0.3614	*Higher & Short‐haul*	0.3165
*Aging & Middle‐class*	0.3592	*Higher & Medium‐haul*	0.4577
*Aging & Business*	0.3635	*Higher & Long‐haul*	0.3293

Last, for passenger segment s, we compare the prediction in the *business as usual* scenario (denoted as $F_{s}^{b}$) with the real number of flights completed by passengers in segment s (denoted as $R_{s}$) to derive the twofold impact of COVID‐19 on the segment, that is, $\frac{F_{s}^{b}-R_{s}}{F_{s}^{b}}$. We compare the prediction in the *pandemic* scenario (denoted as $F_{s}^{p}$) with $F_{s}^{b}$ to derive the impact of COVID‐19 associated with supply restriction, that is, $\frac{F_{s}^{b}-F_{s}^{p}}{F_{s}^{b}-R_{s}}$. The impact of COVID‐19 associated with demand depression is then $\frac{F_{s}^{p}-R_{s}}{F_{s}^{b}-R_{s}}$. Table 6 lists the twofold impact and the impact of each force on each segment.

**TABLE 6:** Twofold impact of COVID‐19, impact associated with supply restriction (S.R.I.) and impact associated with demand depression (D.D.I.) on A1–A9/B1–B9 segments

Age	Purpose of travel	Twofold impact	S.R.I.	D.D.I.
*Young*	*Leisure*	41%	74.7%	25.3%
	*Middle‐class*	40.3%	68.9%	31.1%
	*Business*	38.7%	31.2%	68.8%
*Middle‐age*	*Leisure*	40.7%	39.7%	60.3%
	*Middle‐class*	40.9%	37.1%	62.9%
	*Business*	38.9%	2.2%	97.8%
*Aging*	*Leisure*	39.5%	42.4%	57.6%
	*Middle‐class*	41.7%	49.4%	50.6%
	*Business*	41%	37.3%	62.7%
Average		40.3%	42.6%	57.4%

Tier	Flight length	Twofold impact	S.R.I.	D.D.I.
*Explorer*	*Short‐haul*	40.8%	56.3%	43.7%
	*Medium‐haul*	39.5%	61.2%	38.8%
	*Long‐haul*	47.7%	54.7%	45.3%
*Silver*	*Short‐haul*	46.7%	48.6%	51.4%
	*Medium‐haul*	49.3%	49.4%	50.6%
	*Long‐haul*	45.1%	61.1%	38.9%
*Higher*	*Short‐haul*	36.2%	60.7%	39.3%
	*Medium‐haul*	60.2%	41.7%	58.3%
	*Long‐haul*	33.7%	84.5%	15.5%
Average		44.3%	57.6%	42.4%

## 5 TWOFOLD IMPACT OF COVID‐19 ON AIR FRANCE‐KLM

In the *pandemic* period, the number of executed flights decreased by 88.9% on average, compared to the number in the period from 2019‐03‐01 to 2019‐05‐31. Using our evaluation method and comparing the prediction in the *business as usual* scenario with the real situation, we find that COVID‐19 causes a demand decline of 40.3% on average for passenger segments A1–A9 and a demand decline of 44.3% on average for passenger segments B1–B9. Among A1–A9 segments, the *young & leisure/middle‐class* segments, the *middle‐age & leisure/middle‐class* segments, and the *aging & middle‐class/business* segments have the biggest decrease in demand (each has a decrease of more than 40%), whereas the other segments have slightly smaller decreases (around 38%–39%). Among B1–B9 segments, the *higher & medium‐haul* segment has the biggest decrease in demand, a decrease of 60.2%, whereas the other segments have decreases smaller than 50%.

As explained earlier, the number of unique flights completed by each passenger in a segment cannot be directly compared with the number of flights executed by the airline because a flight can be double counted if more than one passenger in the segment have taken the same flight. However, within the same segment, demand predictions and real demand can be compared because they are both derived at the individual passenger level. Comparing the real situation with the prediction, COVID‐19 is evaluated as having a less severe impact on passenger air transport demand than that if we compare the real situation with the same period last year. This is due to the significant decreasing trend in the 24‐month data (see Figures 2 and 3), and the fact that Air France‐KLM, as well as other European airlines, only started the majority of the flight route restrictions in mid‐March 2020. In addition, it is said that Air France‐KLM maintained a relatively impressive route network during the pandemic; thus, its flight activity has less of a precipitous drop at its lowest points compared to some other carriers (Leigh, 2021).

Comparing the predictions in the two scenarios, the impact of COVID‐19 associated with supply restriction counts for 42.6% of the total demand decline on average for A1–A9 and 57.6% of the total demand decline on average for B1–B9. The impact of COVID‐19 associated with demand depression counts for the other 57.4% for A1–A9 and 42.4% for B1–B9. The result that a large portion of the demand decline is due to supply restriction suggests a promising recovery once Air France‐KLM fully resumes its operations. Separating the two forces of COVID‐19, the respective impact varies between passenger segments. Among A1–A9 segments, the force associated with supply restriction impacted the *young & leisure* segment the most and the *middle‐age & business* segment the least, whereas the opposite result holds for the force associated with demand depression. Specifically, for the *middle‐age & business* segment, the demand depression impact of COVID‐19 counts for 97.8% of the total decrease, whereas the supply restriction impact of COVID‐19 only counts for 2.2%. It means that for this segment of passengers, routes on which they frequently fly were not severely restrained in the *pandemic* period (see Table 5), but they chose not to fly. This result is consistent with the initial belief of the airline.

Compared to other age groups, young people do not have a high risk of severe illness from COVID‐19 and are often seen as taking more risks against social distancing (Reniers, 2020). Thus, the decrease in their flight activities is mainly due to the travel restrictions. People who travel mostly for a business purpose have a high financial standing, and it is argued that this group can easily afford to work from home or do social distancing, compared to people with lower incomes (Holliss, 2020). Thus, for them, it is not the travel restrictions that lowered their travel frequencies, but their low willingness to fly. This is also the reason why in our industry example, for passengers between age 20 and 40, the supply restriction impact of COVID‐19 is bigger than the demand depression impact unless these passengers travel mostly for business. In addition, for passengers within every age range, the demand depression impact of COVID‐19 is the biggest when these passengers travel mostly for business, compared to that when they travel for another purpose. We also find that for passengers between age 61 and 80, the two impacts are relatively equal, unless these passengers travel mostly for business.

Among B1–B9 segments, the impact of COVID‐19 is relatively evenly distributed between the two forces, except for the *higher & long‐haul* segment where the impact associated with supply restriction significantly (84.5%) outweighs the impact associated with demand depression (15.5%). Although there are no noticeable patterns in the results for B1–B9, we can still find that for passengers who often take *long‐haul* flights, the supply restriction impact of COVID‐19 is always bigger than the demand depression impact. This result is reasonable as the majority of the travel restrictions at Air France‐KLM involved *long‐haul* flights. In addition, for passengers at the *explorer* tier, the supply restriction impact is always bigger than the demand depression impact. It can be explained by the same reason behind why *young & leisure* passengers were impacted the most by the supply restriction force of COVID‐19. The different results for A1–A9 and B1–B9 segments also demonstrate the importance of using multiple segmentation plans when applying our evaluation method. It helps identify the differences between passengers in terms of the impact of COVID‐19.

## 6 IMPLICATION FOR AIRLINES AND OTHER INDUSTRIES

The initial request of this research in cooperation with Air France‐KLM is to help them design an effective recovery plan. To do so, we propose a method for separating the two forces of COVID‐19 and evaluating the respective impact on passenger air transport demand for different passenger segments separately. Based on our results, different recovery strategies should be used for different segments. For segments on which the two forces of COVID‐19 have almost equal impacts, for example, the *aging & leisure/middle‐class* segments, both strategies that resume flights and strategies that restore passenger confidence or increase passenger willingness to fly should be deployed. For segments on which the supply restriction impact is bigger than the demand depression impact of COVID‐19, for example, the *young & leisure/middle‐class* segments, the focus of airlines' recovery strategies should be on resuming flights on routes on which these passengers frequently fly. For segments on which the demand depression impact is bigger than the supply restriction impact of COVID‐19, for example, the *young/middle‐class/aging & business* segments, the focus of airlines' recovery strategies should be on restoring passenger confidence or increasing passenger willingness to fly by using targeted promotions. Based on our discussions with multiple airline managers from the passenger relation management department, future research can be done on designing an effective marketing campaign using our evaluation results and data of passenger's response to a specific campaign.

Our evaluation method can also be applied to other industries on which the COVID‐19 pandemic has a similar twofold impact. An example of such industries is the retail industry with a brick‐and‐mortar channel presence where demand dropped also due to the two forces of COVID‐19. First, shops were closed and thus customers cannot visit, and second, customers' desire to go out to shop dropped in times of pandemic. When using our evaluation method, the same four steps apply. First, firms need to segment their customers based on customer demographics and characteristics related to customer purchase habit. Second, based on the availability of the data and the scope of the forecasting problem, firms need to select the appropriate forecasting model. Third, two scenarios need to be simulated. One of the two scenarios represents a normal situation in the pandemic, assuming that customers will follow their behavior pattern. The other scenario represents a pandemic situation in which supply is restrained, considering both the behavior pattern of customers and the impact of supply restriction. Fourth, by comparing the predictions in the two scenarios with each other and with the real situation, firms can derive the twofold impact of COVID‐19 on their business demand and the impact of each force of COVID‐19. Based on the evaluation results, firms can plan recovery strategies effectively.

### 6.1 Limitations and future research

Our study is not exempt from limitations, many of which offer opportunities for future research. First, as our data are from the loyalty program of an airline, we only consider passengers who are registered in the program. Although nonmember passengers are often the ones who rarely fly with the airline, it is interesting to examine whether their behavior changes in the pandemic. If the airline can track the flight history of nonmember passengers, this group of passengers could be included in the future research. Second, our data are from one airline of which the majority of flights depart from EU (particularly, France) and most of passengers are EU residents, and thus, we have not considered the origin of flight or passenger geographic information in passenger segmentation. Such information may be effective in explaining the heterogeneity in passenger behavior. Future research could incorporate data from multiple airlines and examine whether COVID‐19 has a different twofold impact on passengers in different regions. Third, similar to other COVID‐related studies in the early days of the pandemic, our data are limited. At the time of analysis, we had flight data until May 31st, and thus, the pandemic period in our study only lasted 3 months. It would be interesting to investigate how the twofold impact of COVID‐19 on airlines evolves as time proceeds. Future research could also focus on developing advanced clustering methods to segment passengers and developing forecasting models to predict demand in a special scenario.

## Figures and Tables

**FIGURE A2:**
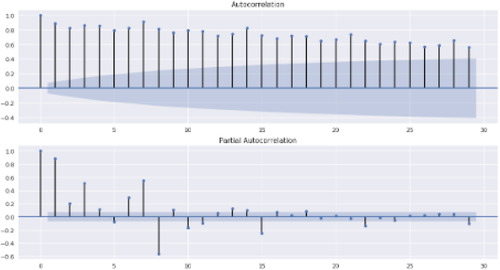
Autocorrelation and partial autocorrelation plots for SARIMA model testing

## Footnotes

1 A flight leg is a flight that maintains the same flight number and aircraft throughout its journey.

2 Flight schedules are mapped out several months in advance by the schedule planning teams at the airlines (Hope, 2017). Given the length of the *pandemic* period in our research that is 3 months starting from March 1st, 2020, it is safe to assume that the flight schedule was made without considering the pandemic.

3 There are four tiers in the *Flying Blue* program: *Explorer*, *Silver*, *Gold*, and *Platinum*. See https://www.klm.com/travel/nl_en/flying_blue/levels/about/index.htm for more details.

## Appendix

**TABLE A1:** Key dates in the dataset

Date	Usage
June 1st, 2018–May 31st, 2020	Length of the *Flying Blue* dataset
March 1st–May 31st, 2020	Pandemic period selected in this study
	Length of the *business as usual* scenario and the *pandemic* scenario
March 1st	Passenger specific data on this date and
	flight specific data till this date
June 1st, 2018–September 30th, 2019	Use time series flight data from this period to test
	the performance of each candidate model
June 1st, 2018–February 29th, 2020	Use time series flight data from this period to predict
	demand for each passenger segment in each scenario
	Use flight data from this period to identify
	passenger flight route choice

**TABLE A2:** TBATS model parameters

Season periods	[14. 30.5]
Seasonal harmonics	[6 1]
ARMA (p, q)	(2, 3)
α	0.764876
β	−0.04858
ϕ	0.949855
γ	[−2.09593603e‐05 2.54518372e‐05 6.34832417e‐06 1.69336316e‐05]
AR coefficients	[6.34832417e‐06 1.69336316e‐05]
MA coefficients	[0.0506144 −0.39585266 −0.07443607]
Seed vector	[9.42639351e+04 −4.44284036e+02 −5.95677756e+02 −1.72887709e+03 −2.59420523e+02 5.44976966e+03 −2.35703158e+02 4.15908728e+03 7.35261057e+00 −6.57202348e+03 −1.81355200e+02 −7.79385936e+03 4.10624044e+01 1.55331475e+03 −2.02719771e+03 1.04546232e+01 0.00000000e+00 0.00000000e+00 0.00000000e+00 0.00000000e+00 0.00000000e+00]

**TABLE A3:** SARIMA model parameters for each passenger segment

Segment	p	d	q	P	D	Q	Segment	p	d	q	P	D	Q
*Young & Leisure*	2	1	3	5	1	1	*Explorer & Short‐haul*	1	1	1	1	1	2
*Young & Middle‐class*	5	1	1	4	1	2	*Explorer & Medium‐haul*	1	1	1	2	1	2
*Young & Business*	1	1	1	4	1	2	*Explorer & Long‐haul*	0	1	1	5	1	0
*Middle‐age & Leisure*	0	1	1	0	1	2	*Silver & Short‐haul*	4	1	3	5	1	1
*Middle‐age & Middle‐class*	4	1	1	3	1	2	*Silver & Medium‐haul*	3	1	1	1	1	2
*Middle‐age & Business*	4	1	3	5	1	1	*Silver & Long‐haul*	1	1	1	2	1	1
*Aging & Leisure*	0	1	1	3	1	1	*Higher & Short‐haul*	1	1	1	5	1	1
*Aging & Middle‐class*	0	1	1	5	1	2	*Higher & Medium‐haul*	0	1	1	5	1	1
*Aging & Business*	3	1	3	5	1	1	*Higher & Long‐haul*	1	1	1	2	1	2

## References

[deci12549-bib-0001] Adikariwattage, V. , de Barros, A.G. , Wirasinghe, S. & Ruwanpura, J. (2012) Airport classification criteria based on passenger characteristics and terminal size. Journal of Air Transport Management, 24, 36–41.

[deci12549-bib-0002] Albers, S. & Rundshagen, V. (2020) European airlines' strategic responses to the covid‐19 pandemic (January‐May, 2020). Journal of Air Transport Management, 87, 101863. 32834690 10.1016/j.jairtraman.2020.101863 PMC7363596

[deci12549-bib-0003] Altay, N. & Green III, W.G. (2006) OR/MS research in disaster operations management. European Journal of Operational Research, 175(1), 475–493.

[deci12549-bib-0004] Amankwah‐Amoah, J. (2020) Note: Mayday, Mayday, Mayday! responding to environmental shocks: Insights on global airlines' responses to covid‐19. Transportation Research Part E: Logistics and Transportation Review, 143, 102098. 33013185 10.1016/j.tre.2020.102098 PMC7522036

[deci12549-bib-0005] Banerjee, N. , Morton, A. & Akartunalı, K. (2020) Passenger demand forecasting in scheduled transportation. European Journal of Operational Research, 286, 797–810.

[deci12549-bib-0006] Bhaskar, A. , Chung, E. et al. (2014) Passenger segmentation using smart card data. IEEE Transactions on Intelligent Transportation Systems, 16(3), 1537–1548.

[deci12549-bib-0007] Briand, A.‐S. , Côme, E. , Trépanier, M. & Oukhellou, L. (2017) Analyzing year‐to‐year changes in public transport passenger behaviour using smart card data. Transportation Research Part C: Emerging Technologies, 79, 274–289.

[deci12549-bib-0008] Brons, M. , Pels, E. , Nijkamp, P. & Rietveld, P. (2002) Price elasticities of demand for passenger air travel: a meta‐analysis. Journal of Air Transport Management, 8(3), 165–175.

[deci12549-bib-0009] Cook, A. , Kluge, U. , Paul, A. & Cristóbal, S. (2017) Factors influencing european passenger demand for air transport. In: Air Transport Research Society World Conference. Air Transport Research Society.

[deci12549-bib-0010] Copperman, R.B. & Bhat, C.R. (2007) Exploratory analysis of children's daily time‐use and activity patterns: child development supplement to US panel study of income dynamics. Transportation Research Record, 2021(1), 36–44.

[deci12549-bib-0011] Craighead, C.W. , Ketchen Jr, D.J. & Darby, J.L. (2020) Pandemics and supply chain management research: toward a theoretical toolbox. Decision Sciences, 51(4), 838–866. 10.1111/deci.12468 PMC7276808 34234384

[deci12549-bib-0012] De Livera, A.M. , Hyndman, R. & Snyder, R.D. (2011) Forecasting time series with complex seasonal patterns using exponential smoothing. Journal of the American Statistical Association, 106(496), 1513–1527.

[deci12549-bib-0013] De Vos, J. (2020) The effect of covid‐19 and subsequent social distancing on travel behavior. Transportation Research Interdisciplinary Perspectives, 5, 100121. 34171016 10.1016/j.trip.2020.100121 PMC7180344

[deci12549-bib-0014] Dia, H. (2001) An object‐oriented neural network approach to short‐term traffic forecasting. European Journal of Operational Research, 131(2), 253–261.

[deci12549-bib-0015] Graham, A. , Kremarik, F. & Kruse, W. (2020) Attitudes of ageing passengers to air travel since the coronavirus pandemic. Journal of Air Transport Management, 87, 101865. 32834691 10.1016/j.jairtraman.2020.101865 PMC7341823

[deci12549-bib-0016] Hamilton, J. (1994) Time series econometrics. Cambridge: Cambridge University Press.

[deci12549-bib-0017] Harrison, A. , Popovic, V. & Kraal, B. (2015) A new model for airport passenger segmentation. Journal of Vacation Marketing, 21(3), 237–250.

[deci12549-bib-0018] Hartigan, J.A. & Wong, M.A. (1979) Algorithm as 136: a k‐means clustering algorithm. Journal of the Royal Statistical Society. Series C (Applied Statistics), 28(1), 100–108.

[deci12549-bib-0019] Hollinger, P. (2020) How coronavirus brought aerospace down to earth. https://www.ft.com/content/3fe8a876‐7d7c‐11ea‐8fdb‐7ec06edeef84 [accessed: 20 January 2021].

[deci12549-bib-0020] Holliss, F. (2020) Working from home is a luxury many renters in the UK can ill afford. https://www.theguardian.com/society/2020/aug/18/working‐from‐home‐is‐a‐luxury‐many‐renters‐in‐the‐uk‐can‐ill‐afford. [accessed: 27 December 2020].

[deci12549-bib-0021] Hope, A. (2017) The Complex Process Behind Your Flight's Schedule. https://www.cntraveler.com/story/the‐complex‐process‐behind‐your‐flights‐schedule. [accessed: 27 March 2021].

[deci12549-bib-0022] Kamarianakis, Y. & Prastacos, P. (2003) Forecasting traffic flow conditions in an urban network: comparison of multivariate and univariate approaches. Transportation Research Record, 1857(1), 74–84.

[deci12549-bib-0023] Kamarianakis, Y. & Prastacos, P. (2005) Space–time modeling of traffic flow. Computers & Geosciences, 31(2), 119–133.

[deci12549-bib-0024] Kriegel, H.‐P. , Kröger, P. , Sander, J. & Zimek, A. (2011) Density‐based clustering. Wiley Interdisciplinary Reviews: Data Mining and Knowledge Discovery, 1(3), 231–240.

[deci12549-bib-0025] Kuljanin, J. & Kalić, M. (2015) Exploring characteristics of passengers using traditional and low‐cost airlines: a case study of Belgrade airport. Journal of Air Transport Management, 46, 12–18.

[deci12549-bib-0026] Kumar, K. , Parida, M. , Katiyar, V. et al. (2013) Short term traffic flow prediction for a non urban highway using artificial neural network. Procedia‐Social and Behavioral Sciences, 104(2), 755–764.

[deci12549-bib-0027] Lee, S. & Fambro, D.B. (1999) Application of subset autoregressive integrated moving average model for short‐term freeway traffic volume forecasting. Transportation Research Record, 1678(1), 179–188.

[deci12549-bib-0028] Leigh, G. (2021) How KLM kept flying during the pandemic. https://www.flightradar24.com/blog/how‐klm‐kept‐flying‐during‐the‐pandemic/. [accessed: 27 March 2021].

[deci12549-bib-0029] Leung, A. , Yen, B.T. & Lohmann, G. (2017) Why passengers' geo‐demographic characteristics matter to airport marketing. Journal of Travel & Tourism Marketing, 34(6), 833–850.

[deci12549-bib-0030] Li, Y. , Wang, X. , Sun, S. , Ma, X. & Lu, G. (2017) Forecasting short‐term subway passenger flow under special events scenarios using multiscale radial basis function networks. Transportation Research Part C: Emerging Technologies, 77, 306–328.

[deci12549-bib-0031] Lippi, M. , Bertini, M. & Frasconi, P. (2013) Short‐term traffic flow forecasting: an experimental comparison of time‐series analysis and supervised learning. IEEE Transactions on Intelligent Transportation Systems, 14(2), 871–882.

[deci12549-bib-0032] Lv, Y. , Duan, Y. , Kang, W. , Li, Z. & Wang, F.‐Y. (2014) Traffic flow prediction with big data: a deep learning approach. IEEE Transactions on Intelligent Transportation Systems, 16(2), 865–873.

[deci12549-bib-0033] Marazzo, M. , Scherre, R. & Fernandes, E. (2010) Air transport demand and economic growth in Brazil: a time series analysis. Transportation Research Part E: Logistics and Transportation Review, 46(2), 261–269.

[deci12549-bib-0034] Mukhopadhyay, S. , Samaddar, S. & Colville, G. (2007) Improving revenue management decision making for airlines by evaluating analyst‐adjusted passenger demand forecasts. Decision Sciences, 38(2), 309–327.

[deci12549-bib-0035] Nai, W. , Liu, L. , Wang, S. & Dong, D. (2017) An emd–sarima‐based modeling approach for air traffic forecasting. Algorithms, 10(4), 139.

[deci12549-bib-0036] Park, J.‐W. (2007) Passenger perceptions of service quality: Korean and Australian case studies. Journal of Air Transport Management, 13(4), 238–242.

[deci12549-bib-0037] Park, Y. & Ha, H.‐K. (2006) Analysis of the impact of high‐speed railroad service on air transport demand. Transportation Research Part E: Logistics and Transportation Review, 42(2), 95–104.

[deci12549-bib-0038] Peterson, B. (2020) What airlines are doing to prove their planes are (extra) clean. https://www.afar.com/magazine/airlines‐assure‐travelers‐their‐airplanes‐are‐extra‐clean‐right‐now. [accessed: 27 December 2020].

[deci12549-bib-0039] Polson, N.G. & Sokolov, V.O. (2017) Deep learning for short‐term traffic flow prediction. Transportation Research Part C: Emerging Technologies, 79, 1–17.

[deci12549-bib-0040] Pournader, M. , Kach, A. & Talluri, S. (2020) A review of the existing and emerging topics in the supply chain risk management literature. Decision Sciences, 51(4), 867–919. 10.1111/deci.12470 PMC7283689 34234385

[deci12549-bib-0041] Reniers, R. (2020) Why do young people take more risks against social distancing. https://www.birmingham.ac.uk/research/perspective/young‐people‐risks‐social‐distancing.aspx. [accessed: 27 December 2020].

[deci12549-bib-0042] Samagaio, A. & Wolters, M. (2010) Comparative analysis of government forecasts for the Lisbon airport. Journal of Air Transport Management, 16(4), 213–217.

[deci12549-bib-0043] Smith, B. (1994) Short‐term traffic flow prediction: neural network approach. Transportation Research Record, 1453, 98–104.

[deci12549-bib-0044] Smith, W.R. (1956) Product differentiation and market segmentation as alternative marketing strategies. Journal of Marketing, 21(1), 3–8.

[deci12549-bib-0045] Suau‐Sanchez, P. , Voltes‐Dorta, A. & Cugueró‐Escofet, N. (2020) An early assessment of the impact of covid‐19 on air transport: just another crisis or the end of aviation as we know it? Journal of Transport Geography, 86. 10.1016/j.jtrangeo.2020.102749 PMC7269949 32834670

[deci12549-bib-0046] Tirachini, A. & Cats, O. (2020) Covid‐19 and public transportation: current assessment, prospects, and research needs. Journal of Public Transportation, 22(1), 1. 10.5038/2375-0901.22.1.1 PMC9468467 36118518

[deci12549-bib-0047] Vlahogianni, E.I. , Karlaftis, M.G. & Golias, J.C. (2005) Optimized and meta‐optimized neural networks for short‐term traffic flow prediction: a genetic approach. Transportation Research Part C: Emerging Technologies, 13(3), 211–234.

[deci12549-bib-0048] Warburg, V. , Bhat, C. & Adler, T. (2006) Modeling demographic and unobserved heterogeneity in air passengers' sensitivity to service attributes in itinerary choice. Transportation Research Record, 1951(1), 7–16.

[deci12549-bib-0049] Williams, B.M. , Durvasula, P.K. & Brown, D.E. (1998) Urban freeway traffic flow prediction: application of seasonal autoregressive integrated moving average and exponential smoothing models. Transportation Research Record, 1644(1), 132–141.

[deci12549-bib-0050] Xie, G. , Wang, S. & Lai, K.K. (2014) Short‐term forecasting of air passenger by using hybrid seasonal decomposition and least squares support vector regression approaches. Journal of Air Transport Management, 37, 20–26.

[deci12549-bib-0051] Zhu, Z. , Sivakumar, K. & Parasuraman, A. (2004) A mathematical model of service failure and recovery strategies. Decision Sciences, 35(3), 493–525.

